# Gastrointestinal Pharmacology

**Published:** 2008-10

**370**

***Emblica officinalis* decreases severity of acute pancreatitis in animal model**

Sidhu S, Pandhi P, Malhotra S, Vaiphei K, Khanduja KL

*PGIMER*, India.

**Background:**

Acute pancreatitis is an inflammatory disease of the exocrine part of the pancreas. It is associated with significant damage to the acinar cells and is associated with high mortality and morbidity. Currently only symptomatic treatment for the disease is available. In experimental studies various agents have been explored for their beneficial effects in acute pancreatitis. Thus the present study was planned with aim to explore whether *Emblica officinalis*, which has strong anti-oxidant properties and anti-inflammatory properties, is beneficial in experimental model of acute pancreatitis.

**Objectives:**

The aim of the present study was to evaluate the effect of *Emblica officinalis* in rat model of acute necrotizing pancreatitis.

**Materials and Methods:**

Acute pancreatitis was induced by L-arginine model. The rats were divided into three groups L-arginine group, L-arginine+ *Emblica officinalis* group and L-arginine+ Vitamin C group. An additional control group was included in which no treatment was given. In each group(n=24) the animals were sacrificed at four time points after induction of pancreatitis i.e. 24 hr, 3 days, 14days and 28 days after acute pancreatitis. Drug administration was started two hours after last injection of L-arginine till the day of sacrifice. *Emblica officinalis* was administered in a dose of 100 mg/kg and vitamin C in a dose of 900mg/kg once daily. At each time point the animals were sacrificed and blood and pancreatic tissue samples were collected. Serum samples were used for the evaluation of serum amylase and lipase levels. Pancreatic tissue was used for the determination of pancreatic amylase, nucleic acid content and total proteins and rate of DNA synthesis. A part of tissue was used for histopathological evaluation.

**Results:**

*Emblica officinalis* was found to be beneficial in acute pancreatitis. Serum lipase levels were significantly lower in *Emblica officinalis+* L-arginine group as compared to L-arginine alone group at day 3 and day 14 after pancreatitis but at 24 hrs there was no significant difference between two groups. The serum amylase levels were significantly lower in *Emblica officinalis+* L-arginine group as compared to L- arginine alone group at day 14and no significant difference was found at 24 hrs and 3 days after pancreatitis. Nucleic acid content of pancreas was more in *Emblica officinalis+* L-arginine group as compared to L-arginine alone group at all time points and similarly rate of DNA synthesis was also more in *Emblica officinalis+* L-arginine group compared to L-arginine alone group. Histopathological examination showed improvement in *Emblica officinalis+* L-arginine group as compared to L-arginine group. Vitamin C was found to be less efficacious then *Emblica officinalis* in all the parameters.

**Conclusion:**

*Emblica officinalis* was found to be beneficial in acute necrotizing pancreatitis. Treatment with *Emblica officinalis* significantly improved the rate of DNA synthesis and total protein content of the pancreas. Histopathological scores were also lower in *Emblica officinalis+* L-arginine group as compared to L-arginine alone group.

**371**

**Anti-ulcer activity of *Punica granatum* L flowers in rats**

Ahmad MP, Alam MS, Ahmad MA, Ahmad S, Najmi AK

Jamia Hamdard, New Delhi – 110062, India.

The male abortive flowers of *Punica granatum* Linn. (family Punicaceae) has been used in different single and compound formulations of Unani system of Medicine for the treatment of gastro intestinal disorders. In the present investigation different physicochemical standards of *Punica granatum* were established and aqueous methanolic extract (AM) of standardized *Punica granatum* was used for its possible gastro protective activity in Wistar rats against different experimental models. Oral administration of AM (490 and 980 mg kg^-1^), significantly reduced the ulcer lesion index produced by alcohol (1 ml each), indomethacin (20 mg kg^-1^) and aspirin (150 mg kg^-1^) p.o., dose dependently in rats. The gastro protective effect of AM was found comparable to or more than that of standard drug (omeprazole 20 mg kg^-1^, p.o.). Further, in pylorusligated rats AM significantly decreased the ulcer lesions, gastric volume and total acidity. It was found to prevent the ulceration by increasing the pH and mucus secretion in pylorus ligated rats. The preliminary phytochemical screening of AM revealed the presence of saponin, tannins and flavonoids, which may be responsible for its gastro protective effect. HPTLC fingerprints of AM showed presence of 12 peaks at different Rf values. The results obtained in present investigation revealed the gastro protective effect of *Punica granatum* and needs a systematic study on this traditional remedy.

**372**

**Evolving process towards drug development from Indian medicinal plants for peptic ulcer disease **

Palit Gautam

Division of Pharmacology, Central Drug Research Institute, Lucknow-226001, India.

Peptic Ulcer Disease (PUD), one of the major gastrointestinal disorders, requires a targeted therapeutic strategy. Clinical evaluations of the potential anti-ulcer drugs have shown incidences of relapses, side effects and adverse drug interaction. In order to overcome these undesirable effects, investigations have been extended for the search of novel molecules from plant sources offering better protection with lower rate of incidences of relapse. Various Indian medicinal plants- *Ocimum sanctum, Allophylus serratus, Desmodium gangeticum, Azadirachta indica, Hemidesmus racemosus, Asparagus racemosus, Musa sapientum* have been reported to offer promising anti-ulcer activity. We have directed our endeavors towards establishing the scientific rational in governing the efficacy of natural products in peptic ulcer, using various animal models to explore their anti-ulcer and ulcer healing properties along with their molecular mechanism of action. In this regard we have focused on *Ocimum sanctum* (OS) which is known to possess various therapeutic properties and identified its potential as an anti-ulcerogenic agent by evaluating against various acute and chronic gastric and duodenal ulcer models in rodents. It significantly reduced free acidity and increased mucin secretion. Moreover, the major gastroprotective effect of OS is mediated through the inhibition of H+ K+-ATPase leading to decrease in acid secretion. Thus with its anti-ulcerogenic and ulcer-healing properties, OS could act as a potent therapeutic agent against PUD.

**373**

**To study the correlation of apoptosis with severity in caerulein induced pancreatitis**

Khosla P, Pandhi P, Malhotra S, Nada R^1^, Rana S^2^, Bhasin DK^1^

Postgraduate Institute of Medical Education and Research, Chandigarh, India.

**Objective:**

The relationship of apoptosis and pancreatic injury during acute pancreatitis is not clear. The aim of the present study was to investigate the relationship between apoptosis and severity in caerulein induced acute pancreatitis in rats.

**Methods:**

Acute pancreatitis was produced in overnight fasted rats (150-250 g) by four intraperitoneal injections of caerulein 50 μg/kg at 1-hour interval. The disease severity was assessed after administering curcumin and rosiglitazone (alone and in combination) 30 minutes prior to caerulein administration. Animals were sacrificed 1 hour after last caerulein injection and severity was assessed by measuring serum amylase, lipase and morphological changes by quantitative scoring of histological sections. Apoptotic Index was measured by TUNEL and Hoechst staining.

**Results:**

Correlation analysis performed between apoptotic index and various indices of pancreatic injury revealed that during edematous acute pancreatitis a positive correlation exists between serum amylase, serum lipase, histological score and apoptotic index. A significant positive correlation suggests that an increase in pancreatic injury is associated with increase in acinar cell loss by apoptosis.

**Conclusion:**

The results of our study suggest that agents with anti-apoptotic properties could be of potential therapeutic value in edematous acute pancreatitis.

**374**

**Hepatoprotective activity of *Colocasia antiquorum* against carbon tetra chloride and paracetamol induced liver injury in rats**

Patil M, Tuse T, Harle U, Yende S, Rajgure D

AISSMS College of pharmacy, Pune, India.

*Colocasia antiquorum* (L.) Schott, (Araceae), is an ancient crop grown throughout the humid tropics. Hepatoprotective effect of the ethanolic extract of corms of *C. antiquorum* was evaluated in paracetamol and CCL_4_ intoxicated rats. The ethanolic extract of *C. antiquorum* at a dose of 500 mg/kg was administered orally for 7 days. The protective effect was evident from serum biochemical parameters and histopathological analysis. Rats treated with extract of *C. antiquorum significantly* (*P*<0.001) inhibited the elevation of Serum Glutamic Oxaloacetic Transaminase (SGOT) and Serum Glutamic Pyruvic Transaminase (SGPT) in Paracetamol and CCl_4_ treated rats. Histopathological changes induced by Paracetamol and CCL_4_ were also significantly (*P*<0.001) reduced by the extract treatment in similar dosage. Pretreatment of 500 mg/kg/day p.o. of the *C. antiquorum* extract for 7 days before paracetamol administration showed significant (*P*<0.001) protection against paracetamol induced elevated levels of SGOT and SGPT as compared to paracetamol group. Extract of *C. antiquorum* significantly (*P*<0.001) decreased the levels of marker enzymes SGOT and SGPT as compared to CCL_4_ group The activity of ethanolic extract of *C. antiquorum* was nearly similar to that of silymarin. Active constituents identified in ethanolic extract were anthocyanins such as cyanidin 3-glucoside, pelargonidin 3-glucoside and cyanidin 3-rhamnoside, in corms of *C. antiquorum* may be responsible for the hepatoprotective activity. It could be concluded that ethanolic extract of *C. antiquorum* possesses significant hepatoprotective properties.

**375**

**Effect of eugenol in stress induced-irritable bowel syndrome and visceral hypersensitivity models**

Shah A, Singh S, Sairam K

Banaras Hindu University, Varanasi, India.

**Introduction:**

Irritable Bowel Syndrome (IBS) is the most prevalent functional gastrointestinal disorder characterized by altered bowel movements and abdominal pain. Almost 10-20% population around the world is suffering from IBS. Although IBS is not a fatal disease, it significantly alters the quality of life. Different theories of pathophysiology namely, stress, infection, visceral hypersensitivity and inflammation, have been proposed and based on these different treatment options have been suggested. Unfortunately, none of them cover the multifactorial component of the disease and hence the present treatment has several limitations. According to literature survey eugenol has not been used for treatment of IBS. Eugenol, an active constituent of clove/cinnamon/Ocimum sanctum, has been reported to have anti-stress, anti-diarrheal, antibacterial, analgesic and anti-inflammatory properties. Hence, eugenol may be effective against IBS as it is targeting most of the causative factors for development of IBS.

**Methods:**

Effect of Eugenol (10, 50, 100 mg/kg, p.o.) on bowel dysfunction was studied using restraint stress induced-IBS model. The effect on visceral hypersensitivity was studied on balloon inflated and irritated colon. For mechanistic study of brain-gut nexus, plasma corticosteroids and anti-oxidant parameters namely, super oxide dismutase, lipid hydroperoxide and catalase were measured in distinct brain regions as well as gut.

**Results:**

Eugenol decreased the symptoms of experimental IBS and visceral hypersensitivity. Eugenol normalized plasma corticosteroid levels elevated after stress-induced IBS. Eugenol also augmented anti-oxidant defenses both in the gut and brain.

**Conclusion:**

Eugenol is effective against experimental models of IBS. The anti-IBS activity of eugenol involves anti-stress and antioxidant effects.

**376**

**Effect of resperidone in stress related mucosal disorder (SRMD) in CRS model**

Saxena B, Devpriya, Sairam K

Banaras Hindu University, Varanasi, India.

**Introduction:**

Stress has been long associated with peptic ulcer disease. Stress is an important etiological factor in about 307–65% of peptic ulcer cases. Although, stress can be one of the important determinants of peptic ulcers, clinically stress-ulcers are a separate entity from common peptic ulcer and are clinically termed as stressrelated mucosal disease (SRMD) which represents a continuum of conditions ranging from stress-related injury (superficial mucosal damage) to stress ulcers (focal deep mucosal damage). SRMD is most commonly seen in critically ill patients in the intensive care unit (ICU) mostly caused by mucosal ischemia. However very few drugs are available for the treatment of SRMD. Thus in our present investigation we study the effect of resperidone in SRMD.

**Methods:**

Effect of Resperidone (0.1, 0.3 and 1 mg/kg, p.o.) acutely and chronically (21 days) on gastric dysfunction was studied using cold restraint stress (CRS) model, by estimating the ulcer index and anti-oxidant parameters namely, super oxide dismutase, lipid hydroperoxide and catalase. For mechanistic study of brain-gut nexus, plasma corticosteroids, hexosamine as well as microvascular blood flow were measured.

**Results:**

Resperidone (0.1 mg/kg, p.o., chronic administration) significantly decreased the ulcer index in CRS induced ulcer. Results also suggested that Resperidone augmented anti-oxidant defenses in the gut. Resperidone normalized plasma corticosteroid levels, hexosamine and microvascular blood flow after stress-induced ulcer to that of control.

**Conclusion:**

Resperidone is effective against experimental models of SRMD. The anti-stress ulcer activity of resperidone involves anti-stress and anti-oxidant effects.

**377**

**Study of the composition of various marketed brands of ORS and compare them with that of WHO formulation**

Yadav VS, Kumar S, Sharma A, Chugh Y, Anand M, Shukla AK, Chaurasia RC, Kastury N

MLN Medical college, Allahabad, India.

**Object:**

To study the composition of various marketed brands of oral rehydration salt (ORS) and compare with that of WHO formulation.

**Materials and Methods:**

Various marketed brands of ORS were studied. Data was collected from various sources and compared with the composition recommended by WHO.

**Observation:**

Total of 18 most popular marketed brands were studied It was found that 9 brands have similar composition as recommended by WHO older formula. Two brands were available according to both older and newer formula of WHO. 3 brands of ORS were found containing dextrose more than the recommended amount and 3 were having rice flour in place of dextrose. 1 brand was found having additional supplements like calcium lactate, magnesium sulfate and sodium acid phosphate. 7 brands were available in the sachets to be dissolved in 1000 ml of water while 4 brands, in the sachets to be dissolved in 200 ml of water. 7 brands were available in the sachet to be dissolved in both 200 ml and 1000 ml of water.

**Conclusion:**

**Introduction** of ORS by WHO in 1969 has simplified the treatment of acute diarrheal diseases. It is available free of cost in government hospitals but many pharmaceutical companies are manufacturing ORS for sale in the market. Regulatory authorities should check the name and logo WHO so that industry may not exploit the opportunities for promoting their branded products.

**378**

**H+/K+- atpase inhibitors: effect on heart: Consequences there off**

Gautam CS, Utreja A, Goel D, Sandhu G

Govt. Medical College and Hospital, Chandigarh, India.

**Objectives:**

To evaluate the effect of the three proton pump inhibitors i.e Omeprazole, Rabeprazole and Pantoprazole on the frog heart rate and amplitude of contraction.

**Materials and Methods:**

The frog heart *in-situ* prepration was set up. The responses were taken with varying doses of the three proton pump inhibitors. Rate and amplitude was tabulated and statistical analysis done using Graphpad statistical software system.

**Results:**

With Omeprazole the rate and amplitude decreased with both 0.2ml and 0.4ml but it was not statistically significant. With Rabeprazole the rate decreased significantly with both 0.2ml and 0.4ml but there was no significant change in the amplitude. With 0.2ml of Pantoprazole the amplitude reduced significantly while no significant change was noted in the rate. But with 0.4ml of Pantoprazole significant reduction in both rate and amplitude of contraction was observed. The experiments showed that there is reduction in the heart rate (bradycardia). There was also occurrence of arrythmias in the form of extrasystoles. But the effects were reversible and normal sinus rhythm was achieved after the drug effect was over. It was observed that the maximum reduction in rate was observed with Rabeprazole (34% with 0.2ml and 45% with 0.4ml) and maximum reduction in amplitude with Rabeprazole (0.2ml, 41%) and Pantoprazole ().4ml, 51%).

**Conclusion:**

The three proton pump inhibitors showed a dose dependant negative chronotropic effect in the frog heart prepration.

**379**

**Gastroprotective potential of *Bergera koenigii* leaves in adult human**

Chaurasia RC^1^, Chugh Y^1^, Chandra S^2^

^1^M.L.N. Medical College, Allahabad; ^2^Diabetic Care Centre, Allahabad, India.

**Background:**

Plant products are associated with mankind since ancient times. These traditional remedies are proved to be effective when all measures get failed, and used for almost all clinical indications with scope and expectation to cure all ailments with no or least side effects. Today people are diverted towards natural therapy due to their availability, effectiveness, prompt therapeutic response and of-course cost.

**Objectives:**

Evaluation of Gastroprotective action of leaves of *Bergera koenigii* (curry patta).

**Methods:**

The observational study was conducted between Jan- Dec 2007 in adults, aged 20-40 yrs, seeking dietary advice for clinically diagnosed gastritis and/or peptic ulcer. A sum total of 76 persons of both sexes were advised to take daily at least 20-25 fresh leaves of *Bergera koenigii*, along with usual bland diet. Follow-up was done monthly for subsequent six months to observe improvement of symptomatology viz. nausea, epigastric pain, flatulence etc.

**Results:**

More than two-third persons (n=58) become total asymptomatic without reoccurrence, while remaining one-third persons showed either partial (n=8) or no improvement (n=10). But interesting part was that bowel habit became regular in almost all persons due to its fibre content.

**Conclusion:**

Conventional medical remedies are even effective in upper G.I. disorder but are accompanied with transient relief and reoccurrence. *Bergera koenigii* leaves contain a volatile oil with peculiar aromatic odour (active principle is a glucaside- koenigin) popularly used for flavouring curries and condiments. Its regular use has a gastroprotective potential to relieve most of gastric symptomatology along with regular bowel habit without reoccurrence.

**380**

**Gastric antisecretary and cytoprotective effect of* Ocimum basilicum***

Kaur P, Suttee A, Kumar V

Lovely Professional University, Phagwara, India.

**Introduction:**

*Ocimum basilicum* belongs to family Labiatae. *Ocimum basilicum* has been reported to possess analgesic, antiinflammatory, anticancer, anti-diabetic, antioxidant and antimicrobial activities.

**Methods:**

In the present study rats were divided into four groups and ulcers were induced by pylorus ligation (PL) method to investigate the possible gastric antisecretary and cytoprotective effect of *Ocimum basilicum*. Group-I was control (1%CMC), Group-II standard (Sucralfate 250 mg/kg), Group-III ethanolic leaves extract of *Ocimum basilicum* (200 mg/kg, p.o.) and Group-IV ethanolic seeds extract of *Ocimum basilicum* (300 mg/kg, p.o.) treated. Ulcers were assessed by ulcer index. Moreover, other parameters such as total acidity, pepsin output, protein content (PR) and total carbohydrates content (TC) were also measured to investigate the possible gastric antisecretary and cytoprotective activity.

**Results:**

Ethanolic extract of seeds and leaves shows the ulcer protective activity of 28.06% and 51.02% respectively as measured by ulcer index. Moreover, the extracts were also found to decrease total acidity, pepsin output, protein content (PR), whereas total carbohydrates content (TC) was increased in PL induced ulcers. Further, increase in TC: PR ratio (a potent marker of mucin secretion) in gastric juice was also observed when compared to standard drug Sucralfate.

**Conclusion:**

From the above study it has been concluded that ethanolic extract of leaves and seeds of *Ocimum basilicum* have cytoprotective and antisecretary effects which may be responsible for the ulcer protective mechanisms.

**381**

**Beneficial effects of l-arginine against diabetesinduced oxidative stress in rats**

Ubgade AM^1^, Kochar NI^1^, Umathe SN^2^

^1^Agnihotri College of Pharmacy, Wardha, M.S., India; ^2^University Department of Pharmaceutical Sciences, Nagpur, M.S., India.

Oxidative stress occurs in diabetics and responsible for genesis of various complications. Arginine level decreases in diabetes. The ability of L-arginine to ameliorate oxidative stress after treatment with alloxan was investigated in rats. Rats (250-300g) were grouped as non-diabetic and diabetic. They were injected intraperitoneally with multiple doses of alloxan (120 mg/kg) to produce experimental oxidative stress characteristics of diabetes and maintained for 8 weeks. They were given daily treatment of L-arginine (0.5mg/ml), through drinking water. Concentration in drinking water supplied to diabetic rats was adjusted (0.15mg/ml on average) to ensure pair feeding. On day 57, rats were decapited and GI tissues were isolated. Plasma glucose levels, glycosylated haemoglobin and various oxidative stress parameters were investigated. Results interpreted and significance was analyzed. Hyperglycemia observed after 3 days of alloxan treatment, associated with a depression of glutathione (GSH) concentration, superoxide dismutase (SOD) and catalase (CAT) activities in the pylorus and ileum (*P*<0.001). Glycosylated haemoglobin and malonyldialdehyde (MDA) levels were significantly elevated (*P*<0.001), indicating increased lipid peroxidation and oxidative stress. L-arginine significantly ameliorated oxidative stress evidenced by lower glycosylated haemoglobin and MDA levels; and a higher level of the endogenous GSH concentration and SOD and CAT activities (*P*<0.001). These effects were paralleled by marked protection and prophylaxis against alloxaninduced hyperglycemia. Thus, exogenously administered L-arginine might improve clinical manifestation of diabetes and decrease the oxidative stress in the gastrointestinal tract. In addition, the study supports the beneficial effects of L-arginine, which might be attributed to its direct, NO-dependent antioxidant capacity and/or NO-independent pathways.

**382**

**Effect of *Oryza sativa* bran oil in various experimental ulcer models**

Panchal S^1^, Goswami SS^2^, Shah MB^2^, Santani DD^2^

^1^Nirma Institute of Pharmacy, Ahemedabad, India, ^2^L. M. College of Pharmacy, Ahmedabad, India.

This study was undertaken to determine the antiulcer activity of O. sativa in swimming stress induced ulcer and pylorus ligation induced ulcer in rats. Extract of *O. sativa bran (rice bran oil*) (1ml/day) was given for four days. On the fourth day pylorus ligation was performed. Ulcer index, acid secretory parameters, mucoprotective parameters and volume of gastric secretion were measured in control and treated pylorus ligated rats. Ulcer index measurement as well as malondialdehyde level, superoxide dismutase, catalase and reduced glutathione assays are performed in stomach homogenate of rats with swimming stress induced ulcer after four days rice bran oild (RBO) (1 ml/day) treatment and compared with control animals. There was a significant decrease in ulcer index, secretory parameters and pepsin activity, and significant increase in mucin activity (TC:PR) with RBO in pylorus ligation induced ulcer model in rats. There was significant reduction in ulcer index as well as the extent of lipid peroxidation and significant increment in preventive antioxidants like superoxide dismutase, catalase and chain breaking antioxidant reduced glutathione. RBO appears to show gastroprotective effect in pylorus ligation induced gastric ulcer model by reduction in secretory parameters and strengthening of gastric mucosal barrier. In swimming stress induced ulcer model, gastroprotective effect of RBO can be associated with its antioxidant potentials.

**383**

**Hepatoprotective and antioxidant activity of *Phaseolous trilobus ait* on bile duct ligation induced liver fibrosis in rats**

Patil SD, Fursule RA

R C Pate Institute of Pharmaceutical Education and Research, Karwand Naka, Shirpur, M S India.

The accumulation of hydrophilic bile acids in the liver is considered to play pivotal role in the induction of hepatic injury. Methanolic (Met) and aqueous (Aet) extract of *P. trilobus* exhibited reduction in elevated level of serum marker enzymes viz AST (aspartate amino transferase), ALT (alanine amino transferase), and LDH (lactate dehydrogenase) with significant reduction in the level of total bilirubin, direct bilirubin, and Hydroxyproline content. Oxidative stress was found to be increased on bile duct ligation; hence, antioxidant property of *Phaseolous trilobus* has also been studied using *in vivo* glutathione estimation, *in vitro* lipid peroxidation and superoxide scavenging assay method. Result showed simultaneous administration of Methanolic and Aqueous extract of *P. trilobus* (100, 200.400mg/kg b.w.) orally once a day for 28 days decreased level of TBARS (Thiobarbituric acid reactive substance), and elevate the level of SOD superoxide dismutase, GSH (glutathione reductase); the protective antioxidants. In conclusion, long term administration of both Met and Aet extracts of *P. trilobus* in rats ameliorated the hydrophilic bile acid induced hepatic injury that probably related to reduced oxidative stress and degree of hepatic fibrosis which could be confirmed by histopathological examination.

**384**

**Ulcer protective effect of ginger oil on aspirin plus pylorus ligation- induced gastric ulcer model in rats**

Sharma H, Khushtar M, Javed K, Bhandari U

Hamdard University, New Delhi, India.

**Introduction:**

Peptic ulcer is a worldwide problem with high prevalence in India. Clinical evaluation of synthetic drugs shows incidences of relapses and drug interactions. Hence, the search for ideal anti-ulcer drug extends to herbal realm which offers better protection and decreased relapse via augmentation of the defensive factors. Dried rhizomes of *Zingiber officinale Roscoe* (Zingiberaceae), having medicinally active volatile oils, accounts for the substantial use of ginger in folk remedies to treat wide range of gastrointestinal ailments. The etiology of peptic ulcer focuses on gamma glutamyl transpeptidase, a brush border enzyme involved in glutathione metabolism and amino acid transfer.

**Objectives:**

To evaluate the protective role of ginger oil on aspirin plus pylorus ligation induced gastric ulceration in rats.

**Methods:**

Study was performed in albino wistar rats of either sex by aspirin (200mg/kg orally for five days) plus pylorus ligation (on sixth day)-induced ulcers. Ginger oil protection was evaluated for two doses (0.5g/kg and 1g/kg per oral) by measuring the ulcer index, serum gamma-GTP levels, acidity of gastric juice and gastric wall mucus thickness.

**Results:**

Ginger oil significantly decreased the mean ulcer index, total acidity, gamma-GTP levels, and increased the mean gastric wall mucus thickness as compared to the aspirin treated plus pylorus ligated rats.

**Conclusion:**

The present investigation provides experimental evidence of the protective action of ginger oil against gastric ulcers induced by aspirin plus pylorus ligation in albino rats.

**385**

**Role of oxidative stress and inflammation in experimentally induced reflux esophagitis in rats**

Singh P, Palit G

Central Drug Research Institute, Lucknow, India.

Reflux esophagitis (RE), a common gastrointestinal disorder has recently been recognized as a serious clinical problem affecting both Asian and western population. RE, a major outcome of increased exposure and/or sensitivity of the esophageal mucosa to gastric contents can result in inflammation and ulceration of esophagus. Other complications of RE include narrowing of esophagus (stricture), Barrett’s esophagus and an increased risk of esophageal cancer. Although reflux of gastric content is an important contributory factor in the development of esophagitis, the exact pathophysiological mechanisms involved in esophageal cell damage are not fully explained by reflux of acid alone. Thus the present study is aimed to evaluate the possible involvement of oxidative stress and inflammation in experimentally induced reflux esophagitis in rats. Tissue Malonaldehyde (MDA), Glutathione (GSH), Superoxide dismutase (SOD) level and mRNA expression of proinflammatory (IL 1 beta, TNF alpha and IL-6) and anti-inflammatory cytokines (IL-10) were assessed as the indexes of oxidative stress and inflammatory responses associated with RE. Induction of reflux esophagitis caused reduction in lipid peroxidation, depletion of total GSH and reduced SOD activity. Moreover gastroesophageal reflux also augmented the inflammatory responses as characterized by upregulated mRNA expression level of TNF alpha, IL 1beta, IL-6 and IL-10. Our results hence demonstrated that oxidative stress and inflammation both have a crucial role in the development of mucosal damage induced due to gastroesophageal reflux. Thus management of oxidative stress and inflammation associated with reflux esophagitis might offer better strategy for development of new therapies with effective clinical responses.

**386**

**Comparative evaluation of different extracts of leaves of *Psidium guajava* linn. for hepatoprotective activity**

Roy CK, Das AK

Krupanidhi College of Pharmacy, Bangalore, India.

The study was designed to evaluate the hepatoprotective activity of different extracts (petroleum ether, chloroform, ethyl acetate, methanol and aqueous) of *P. guajava* in acute experimental liver injury induced by carbon tetrachloride and paracetamol. The effects observed were compared with a known hepatoprotective agent, silymarin (100 mg/kg p.o.). In the acute liver damage induced by different hepatotoxins, *P. guajava* methanolic leaf extract (200 mg/ kg, p.o.) significantly reduced the elevated serum levels of aspartate aminotransferase, alanine aminotransferase, alkaline phosphatase and bilirubin in carbon tetrachloride and paracetamol induced hepatotoxicity. *P. guajava* ethyl acetate leaf extract (200 mg/kg, p.o.) significantly reduced the elevated serum levels of aspartate aminotransferase, alanine aminotransferase and bilirubin in carbon tetrachloride induced hepatotoxicity whereas *P. guajava* aqueous. leaf extract (200 mg/kg, p.o.) significantly reduced the elevated serum levels of alkaline phosphatase, alanine aminotransferase and bilirubin in carbon tetrachloride induced hepatotoxicity. *P. guajava* ethyl acetate and aqueous leaf extracts (200 mg/kg, p.o.) significantly reduced the elevated serum levels of aspartate aminotransferase in paracetamol induced hepatotoxicity. Histological examination of the liver tissues supported the hepatoprotection. It is concluded that the methanolic extract of leaves of *Psidium guajava* plant possesses better hepatoprotective activity compared to other extracts.

**387**

**Evaluation of hepatoprotective activity of ethanolic extract of bark of *Zanthoxylum armatum* dc. in paracetamol induced and carbon tetrachloride (CCL_4_) induced hepatotoxicity in rats**

Sheth AM, Patel KH, Ranawat L, Patel JA

Institute of Pharmacy, Nirma University of Science and Technology, Ahmedabad, India.

**Objectives:**

Drug induced hepatotoxicity is a major problem in drug development. Various herbal drugs have shown promising results in treating liver injury. However, there is a constant need to identify newer natural hepatoprotective agents with greater efficacy, fewer side effects and to explore their potential over synthetic hepatoprotectives.

**Materials and Methods:**

Hepatotoxicity was induced in rats using two different inducing agents namely Paracetamol and Carbon tetrachloride. Male Wistar rats were assigned to six groups containing six animals each. In both the models, Group – I served as normal control. Group – II received either of the hepatotoxins (Paracetamol – 500 mg/kg, p.o. or CCl4 – 0.5ml/kg, i.p.). Group – III received std. drug Silymarin (25 mg/ kg/day, p.o.). An ethanolic extract of bark of *Zanthoxylum armatum* was administered orally at 100, 200 and 400 mg/kg respectively for 28 days, to Group IV, Group V and Group VI respectively, along with either Paracetamol or CCl4. The assessment of hepatoprotective activity was done by evaluating liver function test, antioxidant enzyme activity and histopathology of liver.

**Results:**

Oral administration of ethanolic extract of *Zanthoxylum armatum* DC. produced significant hepatoprotective effect in both the models used. The effect was comparable with the standard drug Silymarin as evident from normalized level of serum enzymes, restoration of liver architecture and significant reduction in lipid peroxidation. A rise in the level of antioxidant enzymes (superoxide dismutase and catalase) was also evident.

**Conclusion:**

In the present study, administration of ethanolic extract of the bark of *Zanthoxylum armatum* was found to have potential hepatoprotective activity.

**388**

**Protective effect of standardized extract of *Ficus racemosa* on experimental reflux oesophagitis in rats**

Ch. V Rao, M Vijayakumar

National Botanical Research Institute, Lucknow, Uttar Pradesh, India.

Protective effect of hydroalcholic extract of the fruits of *Ficus racemosa* Linn (Family: Moraceae) on experimental reflux oesophagitis in rats was investigated. Rats received *F. racemosa* extract (100, 200 mg/ kg × 3days; p.o), omeprazole (30 mg/kg; i.p) and given last dose at 1 h prior to surgery. *F. racemosa* extract at doses 100, 200 mg/kg significantly inhibited the oesophagitis index to 2.32 ± 0.16 (*P* < 0.01) and 1.42 ± 0.14 (*P* < 0.001) respectively, as compare to control group 3.8 ± 0.16. *F. racemosa* extract (200 mg/kg) significantly inhibited the lipid peroxidation (from 0.52 ± 0.04 to 0.38 ± 0.04nmol of malonyldialdehyde (MDA)/mg protein) (*P* < 0.001) and increased in levels of catalase to 27.6 ± 2.2 units of catalase activity/mg protein and superoxide dismutase to 84.4 ± 6.2 units/mg protein (*P* < 0.001). *F. racemosa* extract (100 mg/kg) and omperazole also showed significant inhibition in lipid peroxidation (*P* < 0.001) and enhanced the activities of catalase (*P* < 0.01) and SOD activity. Further, acid and pepsin out put of gastric contents were significantly decreased in treated groups. However, it altered the elevated levels of sialic acid and hexose contents in oesophageal tissue. HPTLC analysis of *F. racemosa* extract showed presence of phenolic compounds gallic acid and ellagic acid, which are known to possess antioxidant properties. The results suggested that antioxidants potential of *F. racemosa* fruit could attenuate the severity of reflux oesophagitis and prevent the oesophageal mucosal damage and this activity may be due to the presence of phenolic compounds.

